# Species composition and risk of transmission of some *Aedes*-borne arboviruses in some sites in Northern Ghana

**DOI:** 10.1371/journal.pone.0234675

**Published:** 2021-06-01

**Authors:** Joannitta Joannides, Mawuli Dzodzomenyo, Faustus Azerigyik, Eudocia Esinam Agbosu, Deborah Pratt, Joseph Harold Nyarko Osei, Rebecca Pwalia, Godwin Kwame Amlalo, Maxwell Appawu, Hayashi Takashi, Shiroh Iwanaga, Andrea Buchwald, Rosemary Rochford, Daniel Boakye, Kwadwo Koram, Kofi Bonney, Samuel Dadzie

**Affiliations:** 1 Department of Parasitology, Noguchi Memorial Institute for Medical Research, University of Ghana, Accra, Ghana; 2 Department of Environmental and Occupational Health, School of Public Health, University of Ghana, Accra, Ghana; 3 Department of Virology, Noguchi Memorial Institute for Medical Research, University of Ghana, Accra, Ghana; 4 Department of Molecular Virology, Tokyo Medical and Dental University, Tokyo, Japan; 5 Department of Environmental Parasitology, Tokyo Medical and Dental University, Tokyo, Japan; 6 Department of Environmental and Occupational Health, School of Public Health, University of Colorado, Aurora, CO, United States of America; 7 Department of Immunology and Microbiology, University of Colorado, Aurora, Colorado, United States of America; 8 Department of Epidemiology, Noguchi Memorial Institute for Medical Research, University of Ghana, Accra, Ghana; National Taiwan Ocean University, TAIWAN

## Abstract

*Aedes*-borne viral diseases mainly Yellow Fever (YF), Dengue (DEN), Zika (ZIK) and Chikungunya (CHK) have contributed to many deaths’ in the world especially in Africa. There have been major outbreaks of these diseases in West Africa. Although, YF outbreaks have occurred in Ghana over the years, no outbreak of DEN, ZIK and CHK has been recorded. However, the risk of outbreak is high due to its proximity to West African countries where outbreaks have been recently been recorded. This study surveyed the mosquito fauna to assess the risk of transmission of Yellow fever (YFV), Dengue (DENV), Chikungunya (CHKV) and Zika (ZIKV) viruses in Larabanga and Mole Game Reserve areas in Northern Ghana. The immature and adult stages of *Aedes* mosquitoes were collected from Larabanga and Mole Game Reserve area. There was a significant (P>0.001) number of mosquitoes collected during the rainy season than the dry season. A total of 1,930 *Aedes* mosquitoes were collected during the rainy season and morphologically identified. Of these, 1,915 (99.22%) were *Aedes aegypti* and 15 (0.22%) were *Aedes vittatus*. During the dry season, 27 *Ae*. *aegypti* mosquitoes were collected. A total of 415 *Ae*. *aegypti* mosquitoes were molecularly identified to subspecies level of which *Ae*. *(Ae) aegypti aegypti* was the predominant subspecies. Both *Ae*. *aegypti aegypti and Ae aegypti formosus* exist in sympatry in the area. All *Aedes* pools (75) were negative for DENV, ZIKV and CHKV when examined by RT- PCR. Three Larval indices namely House Index, HI (percentage of houses positive for *Aedes* larvae or pupae), Container Index, CI (the percentage of containers positive for *Aedes* larvae or pupae) and Breteau Index, BI (number of positive containers per 100 houses inspected) were assessed as a measure for risk of transmission in the study area. The HI, CI and BI for both sites were as follows; Mole Game Reserve (HI, 42.1%, CI, 23.5% and BI, 100 for rainy season and 0 for all indices for dry season) and Larabanga (39%, 15.5% and 61 for rainy season and 2.3%, 1.3% and 2.3 for dry season). The spatial distribution of *Aedes* breeding sites in both areas indicated that *Aedes* larvae were breeding in areas with close proximity to humans. Lorry tires were the main source of *Aedes* larvae in all the study areas. Information about the species composition and the potential role of *Aedes* mosquitoes in future outbreaks of the diseases that they transmit is needed to design efficient surveillance and vector control tools.

## Introduction

Three *Aedes*-borne viral diseases, Dengue, Zika, and Chikungunya were previously known to contribute minimally to global mortality and morbidity [1]. However, over the past five decades the occurrence of these diseases has increased exponentially [2]. Amongst these three diseases, Dengue infection has become the most dangerous and most widespread [3]. In Africa, Dengue has been reported in 34 countries including Togo, Burkina Faso, Côte d’Ivoire, Gabon and others with their urban cities being the most severely affected due to urbanization [4, 5]. Zika virus (ZIKV) which is closely related to Dengue virus (DENV) has circulated in Africa and Asia after its discovery in Rhesus monkeys in the Zika forest of Uganda in 1947 [6]. The disease has received a striking public health attention due to its association with microcephaly and other neurological disorders such as the Guillian Barre syndrome in babies born to infected mothers [7]. The very first outbreak of Chikungunya disease occurred in Tanzania in 1953 and since then, a few outbreaks and sporadic cases were reported mainly in Asia and Africa [8].

The viruses that cause Dengue, Zika and Chikungunya are transmitted by mosquitoes of the *Aedes* species specifically *Aedes aegypti* and *Aedes albopictus*. All three viruses are transmitted either in forest cycle (zoonotic) which involves non-human primates and arboreal mosquitoes [9] or through an urban cycle involving human to humans [9]. Nonetheless, the invasion and the spread of the vectors outside their native range have caused a spillover from the zoonotic transmission cycle to urban transmission cycle involving human primates. Among the *Aedes* species, *Ae*. *aegypti* is known to be the most efficient vector in the transmission of these viruses in urban Africa and some areas in the world [10]. They are efficient vectors because they have adapted to living their entire life cycle in close proximity to humans. They also feed preferentially on human blood even in the presence of other mammals as humans are the most available and stable source of blood [11]. The *Ae*. *aegypti* species is highly susceptible to all three viruses and are efficient in propagating these diseases because to obtain a single blood meal, they bite several humans hence transmit the virus to multiple hosts [1]. *Ae*. *albopictus*, also known as the “Asian tiger” mosquito, has become an important vector in many regions as it complements the role of *Ae*. *aegypti* in a lot of places. The spread of this invasive species of *Aedes* mosquitoes is increasing rapidly. It has become effective in transmitting some of these *Aedes*-borne viruses, especially CHIKV. This is due to, among other factors, the E1-A226V mutation in the Eastern/Central/South African genotype which has conferred an enhanced transmission of the virus in *Ae*. *albopictus* [8] in many parts of the world. There are no vaccines for Dengue, Zika and Chikungunya infections, vector control is the most viable approach to controlling these diseases [12, 13].

Ghana has previously not recorded outbreaks of Dengue, Chikungunya and Zika virus infections but the country is at risk due to its proximity to West African countries where recent outbreaks had occurred [14]. Yellow fever (YF) outbreaks had occurred in Ghana with major outbreaks in northern sector between 1969 and 1980 with a total of 268 deaths [15], however, the presence of a vaccine against YF has substantially mitigated the risk and outbreaks. Recent cases of YF which claimed 2 lives occurred from October 2011 and February 2012 [16]. Recent studies have established previous exposure to Dengue virus among some diagnosed malaria patients in some urban areas in Ghana [17]. A recent study confirmed the presence of Dengue virus in the blood of two children in the Greater Accra region. Further investigations showed that these children had not travelled outside the country and therefore it can be postulated that the infection was acquired locally [18]. Local transmission may also mean that they may have acquired the infection from the bite of an infected *Aedes* mosquito. There is however very limited information on the distribution, abundance and risk of transmission of some *Aedes*-borne arboviruses such as DENV, CHKV and ZIKV in the country.

The Mole Game Reserve located near Damongo in Northern Ghana has been previously described as a high-risk area for transmission of viral hemorrhagic fevers because of the presence of high population of *Ae*. *aegypti* in the Damongo area [19]. However, the southern part of Ghana has been found to be a low-risk area [20]. The Damongo and Mole Game Reserve area have been a hotspot for Yellow fever outbreaks in recent times. The area is also a tourist site that receives many visitors from different parts of the world including some areas endemic for DENV, CHKV and ZIKV. The presence of travelers in and around the Mole Game Reserve where primates such as monkeys serve as reservoir of these wild viruses makes it a potential area for future outbreaks of DENV, ZIKV and CHKV. It is therefore highly imperative to conduct entomological studies in this area to better inform the relevant authorities about possible outbreaks and plan control strategies against the vectors. This study was aimed at investigating the species composition and assessing the risk of transmission of arboviral diseases around some yellow fever hotspot areas in Northern Ghana.

## Materials and methods

### Study area and design

**Fig 1:**
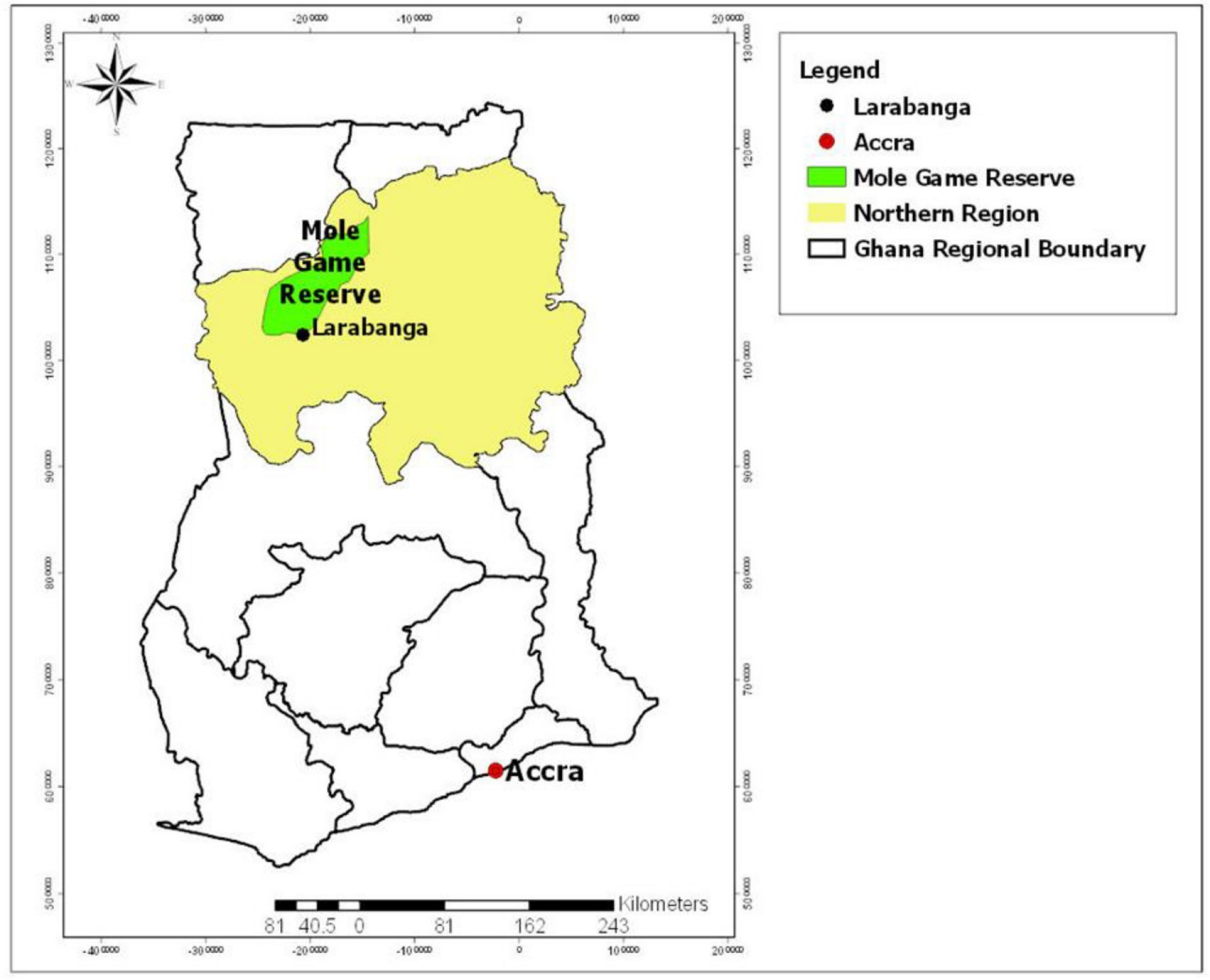
Map of Ghana showing the study sites. Map was developed using shape files of Ghana from the public domain repository of Natural Earth (http://www.naturalearthdata.com/) and built with ArcGIS.

A cross-sectional entomological study was carried out in the Mole Game Reserve quarters area and Larabanga in the Northern region of Ghana (Fig 1). The survey was carried out in the rainy season (October 2018) and the dry season (March 2019). Both areas are in the West Gonja district. The Mole Game Reserve area is a guinea savannah ecological zone with latitude and longitude of 9˚30′ 0′′ N 2′0′ 0′′ W with an elevation of 200 meters. The Game Reserve is home for about 400 species of animals including elephants, chimpanzees, birds and others. The Reserve is more rural and isolated from any major urban town with employees living in quarters inside the game reserve with close proximity to monkeys and other wild animals. It attracts a great number of tourists both, nationally and internationally who normally stay in a motel or hotel located inside the Game Reserve. Larabanga (9˚ 13′ 0′′ N 1˚ W 51′ 0′′) is the nearest semi-urban town about 44 km away from the Mole Game Reserve with a population of about 5000 people. The village is known for its Sahelian mosque which is the oldest mosque in all of Ghana and if possible West Africa. Tourists who visit the game reserve usually visit the Larabanga mosque due to its proximity. These sites were selected sampled based on the potential of zoonotic infections and also previous histories of outbreaks of Yellow fever and suspected viral haemorrhagic fevers which are caused by flavivirus.

Permission was sought from the Game and Wild Life Division of the Ghana Forestry commission to enable us sample from the Mole Game Reserve are and Larabanga community.

### Mosquito collection and larval survey

Adult mosquitoes were sampled using baited BG-Sentinel Biogents Mosquito traps with a combination of ammonia, lactic acid and caproic acid as lure and sweep nets to capture *Aedes* mosquitoes resting on vegetation outdoors. The BG-Sentinel Traps were distributed uniformly across four quadrats of the area around the Game Reserve. A total of twelve (12) BG-Sentinel traps (3 traps per quadrat) were set daily at 06:00h around the Game Reserve and Larabanga and collected 12h later for a period of 5 days for each season around areas shaded by trees. A total of 12 sweep nets were used to sample from vegetation around the Game Reserve and Larabanga at every sampling period. Adult mosquitoes collected were grouped according to sex and put in 2 ml vials and labelled with information on place and date of collection after which they were immediately stored in liquid nitrogen to keep the integrity of the genetic material. Larvae were collected from selected houses which were in close proximity to breeding sites in and around the Mole Game Reserve as well as in Larabanga using ladles, dippers, pipettes and buckets. Larval sampling was also done from natural breeding sites such as tree holes, rock pools etc. Larvae collected were grouped and stored in appropriately labelled falcon tubes containing RNA-later to keep the integrity of the genetic material and also to prevent decomposition that would change morphological characteristics and make identification difficult. All samples were transferred to the Noguchi Memorial Institute for Medical Research-Vector laboratories where they were morphologically identified to species level using identification keys [21].

Houses and containers inside and around houses were inspected for the presence of *Aedes* larvae and pupae. The data from the larval survey was used to calculate the larval indices which are a surrogate marker for the risk of transmission of Arboviruses. During the survey, sites where *Aedes* samples were collected and the houses that were inspected for immature stages of *Aedes* larvae were geo-referenced using Geographical Positioning System (GPS) and the data spatially displayed on a map.

### Molecular identification of *Aedes aegypti* species

DNA was extracted from the legs and last abdominal segment of each *Aedes* mosquito sample using the DNeasy Blood and Tissue Qiagen kit (QIAGEN Cat No 69504) following the protocol described by the manufacturer and previous studies [22]. DNA extracted was amplified using Random-amplified polymorphic DNA polymerase chain reaction (RAPD-PCR). This involved amplification of random segments of *Aedes* mosquito DNA using a 10 base pair primer B3 [23]. A volume of 20 μl was used for the PCR reaction. This contained 13.2 μl of DNase free water, 2 μl of 10X reaction buffer, 0.6 μl of 50 μM Magnesium Chloride, 0.5 μl of 10 μM dNTPs, 0.5 μl of 10 μM B3 primer, 0.25 μl of μM Taq Polymerase and 3 μl of DNA template. The PCR cycling conditions includes an initial denaturation step at 94 ˚C for 4 min, followed by 45 cycles consisting of 94 ˚C for 1 min, 35 ˚C for 1 min and 72 ˚C for 2 min and a final extension step at 72 ˚C for 5 min. The PCR products were electrophoresed at 100 volts for 1 hour and 15 minutes on 1.5% agarose gel stained with ethidium bromide. The PCR products were run alongside 1 kb ladder and visualized using a trans-illuminator [22].

### Processing of mosquitoes for viral detection

Mosquitoes were grouped in pools of 30 and homogenized in Minimum Essential Medium (MEM) containing Earle’s Salts, 2% L-glutamine, supplemented with 10% heat inactivated FBS and Penicillin-Streptomycin. The homogenization was done using hand held automatic homogenizers and pistles. The homogenate was centrifuged at 12000 rpm for 3 min and the supernatant divided into two new tubes. One tube was labeled for storage at -80 ˚C and the other used for RNA extraction.

### Viral detection using Trioplex real-time RT-PCR assay

Total RNAs of the samples were extracted using the QIAmp Viral Mini Kit according to the manufacturer’s protocol. The Trioplex Assay for detection of DENV, CHIKV and ZIKV were done using AgPath-ID RT-PCR kit following the protocol by Centres for Disease Control and Prevention [24]. The RT-PCR reaction of 20 μl was made up of 0.5 μl Nuclease free water, 12.5 μl of 2X RT Buffer, 0.5 μl of DENV mix, 0.5 μl of CHIKV mix, 0.5 μl of ZIKV mix, 0.5 μl of enzyme mix and 5 μl of RNA template. The Real-Time Polymerase Chain Reaction (RT-PCR) cycling conditions includes a reverse transcription step at 50 ˚C for 30 min, a hot start step at 95 ˚C for 2 min, a denaturation step at 95 ˚C for 15 sec and an annealing and extension step at 60 ˚C for 1 min for 45 cycles. This was done using a 7500 fast real-time machine. Nuclease free water was used as the negative control and inactivated DENV, ZIKV and CHIKV were used as positive controls.

### Data analysis

Three indices were used to assess *Aedes* mosquito density in the various collection sites. These were House index (HI), Container index (CI) and Breteau index (BI). HI was expressed as the percentage of houses infested with *Aedes* larvae; CI as the percentage of containers infected with larvae or pupae; and BI as the number of positive containers per 100 inspected houses. The risk of transmission of *Aedes*-borne viruses at the study site was estimated using the WHO criteria. The criteria indicates that the risk of *Aedes*-borne viruses is considered to be high when BI, HI, and CI exceed 50, 35 and 20 respectively; the density of *Ae*. *aegypti* is considered to be sufficient to promote an outbreak of *Aedes-*borne viral disease when BI is between 5 and 50; an area is unlikely for *Aedes*-borne virus transmission to occur when BI, HI and CI is less than 5, 4 and 3 respectively. The proportions of mosquitoes by species, season and site were also calculated using Fishers exact test.

## Results

**Fig 2:**
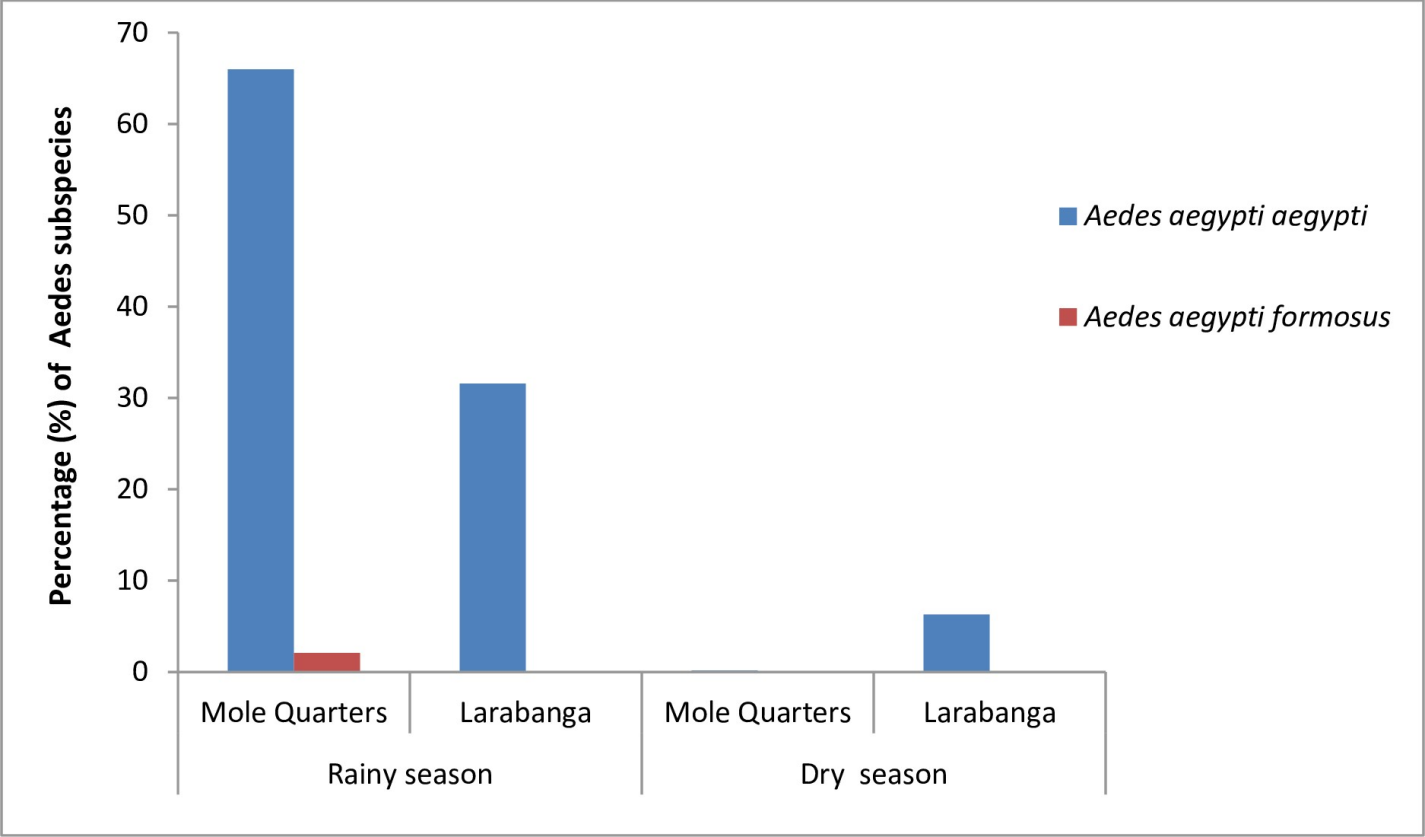
Seasonal distribution of *Aedes aegypti* sub-species in the study areas.

A total of 1957 *Aedes* mosquitoes were collected from the study sites of which 906 (46.3%) were adult *Aedes* mosquitoes and (1051) 53.7% were *Aedes* larvae. Out of the 906 adults collected, a significant (P<0.001) proportion (92.7%) was captured from the Sweep Nets compared to the BG-sentinel traps especially in the rainy season. In the rainy season 1930 *Aedes* mosquitoes were collected and in the dry season 27 *Aedes* mosquitoes were collected (Table 1). Fishers exact test indicated that *Aedes* mosquitoes collected during the rainy season were significantly higher than *Aedes* mosquitoes collected during the dry season (P < 0.001). *Aedes aegypti* was identified in both sites during the rainy and dry seasons. *Aedes vittatus* was only identified in the Mole game reserve area during the rainy season. No *Ae*. *albopictus* was detected in both areas during the study. A total of 415 *Ae*. *aegypti* mosquitoes collected from both sites during the rainy season and dry season were molecularly identified to subspecies level. The two subspecies, *Ae*. *aegypti aegypti* and *Ae*. *aegypti formosus* were identified in Mole game reserve during the rainy season (Fig 2). *Aedes aegypti aegypti* was also identified in Larabanga during both seasons.

**Table 1:** Number of *Aedes species* identified for Mole Game Reserve and Larabanga during the rainy and dry seasons.

Study site	*Aedes* species collected				
	*Aedes aegypti*		*Aedes vittatus*		Total N (%)
	Rainy season N (%)	Dry season N (%)	Rainy season N (%)	Dry season N (%)	
Mole game reserve (Quarters)	1771 (90.5)	1 (0.1)	15 (0.8)	0	1787 (91.3)
Larabanga community	144 (7.4)	26 (1.3)	0	0	170 (8.7)
Total	1915	27	15	0	1957

### *Aedes* larval indices

Larval indices (BI, HI and CI) were estimated for Mole game reserve and Larabanga during the rainy season and dry season. 167 households were surveyed during the rainy season and 206 households were surveyed during the dry season. All three indices were high during the rainy season than the dry season. Observed larval indices were higher in Mole quarters compared to Larabanga. All larval indices (HI, CI and BI) for Mole during the rainy season were above the WHO threshold. HI and BI were above the WHO threshold for Larabanga during the rainy season (Table 2). During the survey, 242 containers that contained water were observed of which 18.2% were positive for *Aedes* larvae during the rainy season. In the dry season, 220 containers that contained water were inspected of which 2.3% were positive for *Aedes* larvae. Overall, 45.5% of households were positive for *Aedes* larvae in Mole game reserve area and 36.4% for Larabanga during the rainy season. In this study, discarded car tires and water storage containers had high positivity rate of over 80% for *Aedes* larvae compared to the other containers inspected during the rainy season followed by earthen ware pots. However, it was observed that the earthen ware pots were the most positive for *Aedes* larvae during the dry season. There were no positive containers observed in Mole game reserve area during the dry season. In the rainy season houses positive for *Aedes* larvae were clustered just around the breeding sites. It was observed that most of the breeding sites were extremely close to households in the Mole area. There were no positive houses and breeding sites during the dry season. The same pattern was observed during the rainy season in the Larabanga area. In the dry season there were no breeding sites observed however, there were a few households that were positive for *Aedes* larvae.

**Table 2:** *Aedes* mosquito larval indices and WHO threshold for transmission risk of *Aedes*-borne viral diseases in Mole Game Reserve and Larabanga.

Larval indices for study areas	Rainy season	Dry season	WHO Threshold for transmission risk of VHF’s
**Mole Quarters**			
House index	42.1*	0.0	4–35 or above
Container index	23.5*	0.0	3–20 or above
Breteau index	100.0*	0.0	5–50 or above
**Larabanga**			
House index	39.0*	2.3	4–35 or above
Container index	15.5	1.3	3–20 or above
Breteau index	61.0*	2.3	5–50 or above

*above the WHO threshold, VHF = Viral Hemorrhagic Fever.

### Detections of DENV, ZIKV and CHIKV in *Aedes* samples

Overall 75 pools consisting of 1957 *Aedes* mosquitoes were tested. These included both males and females. There were 66 pools for *Ae*. *aegypti* and 2 pools for *Ae*. *vittatus* for the rainy season. *Ae*. *aegypti* (7 pools) collected in the dry season were also used for detection of *Aedes*-borne arboviruses. All pools were negative for DENV, ZIKV and CHKV.

## Discussion

In this study, mosquitoes were collected from the Mole quarters and from Larabanga during the rainy and dry seasons. Larval sampling using the dipping method yielded high numbers of *Aedes* mosquitoes during the rainy season. The BG-Sentinel traps were less effective in this area during the rainy season because the traps were disrupted by rain and the wind and this affected the number of mosquitoes collected. There were high numbers of adult *Aedes* mosquitoes collected during the rainy season using the sweep nets. The abundance of *Aedes* populations is controlled by rainfall because it supports the development of additional breeding sites, hatching of eggs, growth of vegetation cover and cool shaded environment for the development of immature stages and high relative humidity [25]. *Aedes* mosquitoes collected during rainy and dry season in the study sites were predominantly *Ae*. *aegypti*. This may be due to the presence of discarded water holding containers and water storage containers around the human settlement which is the preferred breeding site for *Ae*. *aegypti* female mosquitoes to lay their eggs [26]. *Aedes aegypti* are also known to live in close proximity to humans and they prefer human blood even in the presence of other animals [11]. This is evident in the spatial maps for the sites, which shows the proximity to major breeding sites to houses positive for *Aedes* larvae. This characteristic increases the potential of transmission of arboviruses among humans. Furthermore, although the dry preceded the rainy season, the high numbers of *Ae*. *aegypti* mosquitoes recorded during the rainy season may be due to the availability of potential breeding sites provided by the rains. These species of mosquitoes have their eggs being able to withstand desiccation and only hatch when conditions are favorable [27]. The presence of the major vector for the transmission of DENV, CHIKV and ZIKV in the study areas indicates the possibility of future outbreaks. The second *Aedes* species observed was *Ae*. *vittatus*. This species was only identified in the Mole game reserve during the rainy season. This species is usually predominant in forest and savanna areas which is a characteristic of the Game Reserve area [28] and were found breeding mainly in tree holes in the Game Reserve. The distribution of the various species in the area could be due the ecology of the area. The *Ae*. *vittatus* has been incriminated as a vector for yellow fever in various parts of Africa [10, 28, 29]. DENV, ZIKV and CHKV have been isolated from this species indicating that it has the potential to replicate and transmit these viruses experimentally [29]. No *Ae*. *albopictus* was collected in the area during the study. A previous study [19, 22] in the same area also did not find *Ae*. *Albopictus* in the samples collected. However, a survey [20] in southern Ghana detected the species in samples collected although in relatively low numbers. This may be due to the proximity of the southern site to a landing port which has been found to be the main route of importation of the species in many countries.

The study identified *Ae*. *aegypti aegypti* (*Aaa*) as the dominant subspecies in both study sites during the rainy and dry seasons. This species is known to be the “domesticated” form of *Ae*. *aegypti* and it is closely associated with and dependent on human habitats. This may be the reason for their presence in both sites. However, the other subspecies, *Ae*. *aegypti formosus* (*Aaf*) was identified only in the Mole game reserve. *Aaf* are the ancestral African type of *Aedes aegypti* that prefer nonhuman mammals as a blood source [30]. The game reserve is therefore a suitable place for their survival due to the presence of a wide variety of nonhuman mammals. These species were collected from breeding sites which were close to human settlements in the game reserve area. Their presence in these areas may be due to the presence of monkeys and warthogs around human settlements.

In assessing the risk of transmission of YF, DENV, ZIKV and CHIKV, water holding containers around the study sites were surveyed for the presence or absence of immature stages of *Aedes* mosquitoes. The presence of water holding containers around the study sites allows the breeding of *Aedes* larvae thereby increasing the population of *Aedes* mosquito and the associated risk for arbovirus transmission [31, 32]. During the rainy season the larval indices HI, BI and CI for Mole game reserve were above the threshold values for the WHO criteria, but higher than those of a similar study in Damongo which is about 15 km from the Mole Game reserve [19] where the HI, BI and CI were 87.7, 180.9 and 44.8 respectively. These high observed values for Mole Game Reserve indicates a high risk of transmission if Dengue, Chikungunya and Zika cases become established in the area. For Larabanga, there were high values recorded for HI and BI during the rainy season. However, CI was within the threshold values. In the dry season, the larval indices for both sites were extremely low compared to the rainy season. These observations are in agreement with other studies [22, 32]. However, this contrasted with results from another study where it was shown that larval indices were higher in the dry season compared to the rainy season in the Northern region of Ghana [19]. In the dry season, the Mole and Larabanga communities received pipe borne water two days in a week and most water storage containers are cleaned regularly. This may have reduced the numbers of *Aedes* mosquitoes in the area hence the low larval indices observed in those areas. The individuals in Larabanga also relied on water from the dam which was accessed daily due to the higher demand for water during the dry season. Therefore, water was not stored for longer periods during this season. In the Mole quarters area, larval indices were low during the dry season. It was observed that *Aedes* larvae were mostly found in outdoor containers than in indoor containers. This has also been observed in some studies [32, 33] Individuals preferred storing water indoors rather than outdoors because the monkeys in the game reserve area disturbed their water collections and items when stored outdoor. Other studies showed that *Aedes* mosquitoes were breeding indoor rather than outdoor [23, 34] and this may be because water containers stored outdoor are well covered, preventing *Aedes* mosquitoes from laying their eggs inside these outdoor water storage containers.

During the study, discarded tires and water storage containers were the major source of *Aedes* larvae in both areas. Discarded tires usually collect water and tend to harbor *Aedes* larvae without interruption. This makes it an ideal place for *Ae*. *aegypti* mosquitoes to breed undisturbed. Earthen ware pots are usually used to store drinking water. Due to the cool temperature, humidity and reduced light, it serves as a suitable environment for *Aedes* mosquito breeding. All pools of *Aedes* mosquitoes from Mole game reserve and Larabanga analyzed for DENV, ZIKV and CHKV were negative. This is in accordance with another study in the area where all *Aedes* mosquitoes collected from various sites in the Northern region of Ghana were negative for flaviviruses [19]. This may be due to the relatively low number of adult *Aedes* mosquitoes collected from the area. It is recommended a longitudinal study in the area in future that may increase the adult sample size enough to be able to detect any virus circulating in the mosquitoes. *Aedes aegypti formosus* was one of the subspecies identified in the study area. This species have also been recently documented in the area in relatively higher numbers [22, 35]. This species has been shown to be refractory to DENV especially DENV serotype 2 [36]. This may be the reason why these viruses were not detected and possibly why there are no outbreaks of these diseases in Ghana although the area is noted for YF outbreaks. Vector competence studies is recommended in the area to determine the role of the *Ae*. *aegypyi aegypti* and *Ae*. *aegypyi formosus* subspecies in the transmission of DEN and other arboviruses in the area.

## Conclusions

In this study, *Ae*. *aegypti* and *Ae*. *vittatus* were the two species identified around the Mole game reserve. The predominant species of *Aedes* identified in both study sites was *Ae*. *aegypti* which was documented in the rainy season and dry season. Among the *Aedes aegypti* species identified, majority were *Ae*. *aegypti aegypti*. The risk of transmission for DENV, ZIKV and CHKV in the study areas as shown by the larval indices were high during the rainy season than the dry season. This study found that discarded tires and earthen ware pots were the preferred breeding habitats for these species. The study also showed that *Aedes* mosquitoes were breeding in close proximity to human habitation and all *Aedes* mosquito pools were however negative for DENV, ZIKV and CHKV.

## Supporting information

S1 Appendix Larval indices for Mole game reserve (Quarters) and Larabanga during the rainy season and dry season. (DOCX)

S1 Data Rainy and dry season supplemental data. (XLSX)

## Abbreviations

DENV: Dengue Virus
CHIKV: Chikungunya Virus
ZIKV: Zika Virus
CDC: Centers for Disease Control and Prevention
WHO: World Health Organization
PCR: Polymerase Chain Reaction
DNA: Deoxyribonucleic Acid
RNA: Ribonucleic Acid
RAPD-PCR: Random Amplified Polymorphic DNA Polymerase Chain Reaction
RT-PCR: Reverse Transcriptase Polymerase Chain Reaction
HI: House Index
BI: Breteau Index
CI: Container Index
GPS: Global Positioning System
NMIMR: Noguchi Memorial Institute for Medical Research
GIS: Geographical Information System
