# Supplementary material for: Species composition and risk of transmission of some Aedes-borne arboviruses in some sites in Northern Ghana
Source: PLoS One. 2021 Jun 1;16(6):e0234675. doi: 10.1371/journal.pone.0234675 (PMC8168856; doi:10.1371/journal.pone.0234675)
Supplement: S1 Appendix — (DOCX) [file pone.0234675.s001.docx]

## S1 Appendix. Larval survey for Aedes mosquitoes in Mole game reserve (Quarters) and Larabanga during the rainy season and dry season.

|  | Mole game reserve(quarters) | | Larabanga | |
| --- | --- | --- | --- | --- |
|  | Rainy season | Dry season | Rainy season | Dry season |
| Households inspected | 123 | 123 | 44 | 86 |
| Positive households, n (%) | 56(45.5%) | 0 | 16(36.4%) | 20(25%) |
| Containers inspected | 81 | 64 | 161 | 156 |
| Positive containers, n (%) | 19(23.5%) | 0(0%) | 25(15.5%) | 5(3.2%) |
